# Micro-CT 3D imaging reveals the internal structure of three abyssal xenophyophore species (Protista, Foraminifera) from the eastern equatorial Pacific Ocean

**DOI:** 10.1038/s41598-018-30186-2

**Published:** 2018-08-14

**Authors:** Andrew J. Gooday, Dan Sykes, Tomasz Góral, Mikhail V. Zubkov, Adrian G. Glover

**Affiliations:** 1 National Oceanography Centre, University of Southampton Waterfront Campus, Southampton, UK; 2 Imaging and Analysis Centre, Natural History Museum, London, UK; 3 Life Sciences Department, Natural History Museum, London, UK; 4 Present Address: Henry Moseley X-ray Imaging Facility, School of Materials, University of Manchester, Manchester, UK; 5 Present Address: Scottish Association for Marine Science (SAMS), Scottish Marine Institute, Oban, Argyll UK

## Abstract

Xenophyophores, giant foraminifera, are distinctive members of the deep-sea megafauna that accumulate large masses of waste material (‘stercomare’) within their agglutinated tests, and organise their cells as branching strands enclosed within an organic tube (the ‘granellare’ system). Using non-destructive, three-dimensional micro-CT imaging we explored these structures in three species from the abyssal eastern Pacific Clarion-Clipperton Zone (CCZ). In *Psammina* spp., the low-density stercomare occupied much of the test interior, while high-density granellare strands branched throughout the structure. In *Galatheammina* sp. the test comprised a mixture of stercomare and test particles, with the granellare forming a web-like system of filaments. The granellare occupied 2.8–5.1%, the stercomare 72.4–82.4%, and test particles 14.7–22.5%, of the ‘body’ volume in the two *Psammina* species. The corresponding proportions in *Galatheammina* sp. were 1.7% (granellare), 39.5% (stercomare) and 58.8% (test particles). These data provide a potential basis for estimating the contribution of xenophyophores to seafloor biomass in areas like the CCZ where they dominate the megafauna. As in most xenophyophore species, the granellare hosted huge numbers of tiny barite crystals. We speculate that these help to support the extensive granellare system, as well as reducing the cell volume and lightening the metabolic burden required to maintain it.

## Introduction

It has been known for more than 100 years that xenophyophores, a distinctive group of large agglutinated foraminifera confined to deep-sea habitats^1^, are particularly diverse in the eastern equatorial Pacific^2–4^. This region falls within the Clarion Clipperton Zone (CCZ), an enormous tract of seafloor characterised by extensive fields of polymetallic nodules that are of current commercial interest^5–7^. The International Seabed Authority (ISA), a body set up under the United Nations Convention on the Law of the Sea to regulate exploitation of seabed resources beyond national jurisdictions, has licensed areas of seafloor in the CCZ for exploration and prospecting by companies and other bodies with interests in seabed mining. The licences require companies to conduct biological and environmental surveys prior to any future mining activities. In the UK-1 and adjacent Ocean Mineral Singapore (OMS) areas, both located in the eastern part of the CCZ, this requirement is being met by the Abyssal Baseline (ABYSSLINE) project. An ABYSSLINE survey of megafaunal organisms in seafloor photographs from the UK-1 area, revealed that xenophyophores are the dominant visible megafaunal organisms^8^. Similar results were obtained in the Russian license area in the central CCZ^9^. A recent study^4^ based on specimens collected in core samples recognised a total of 36 morphospecies in the UK-1 and OMS license areas, a number that represents a substantial increase in the global diversity of these protists. As well as being important in their own right, xenophyophores provide habitat structure and other ecosystem services that are utilised by many other deep-sea organisms^10^.

In addition to the large size of their agglutinated tests, xenophyophores display a number of distinctive features that set them apart from other foraminifera. The cell body is organised as a system of strands and threads enclosed by a thin-walled organic tube, these two elements together constituting the ‘granellare system’. The cytoplasm itself contains numerous small crystals (‘granellae’), a few microns in size, that are probably obtained from the surrounding environment. In the vast majority of xenophyophores, including those from the CCZ, the crystals are of barite (barium sulphate)^11^. However, the fact that high-density titanium-bearing minerals dominate in one species from an Atlantic submarine canyon^12^ suggests that xenophyophores accumulate barite because it is a heavy mineral. The test interior also contains the stercomare, dark grey masses of waste pellets (stercomata) contained within an organic membrane that usually occupy a larger volume than the granellare^1^. The stercomata consist mainly of small particles that appear plate-like in transmission and scanning electron microscope images^1,13^ and are probably clay minerals. It seems likely, therefore, that xenophyophores feed by gathering sediment particles, either from the surrounding seafloor, or from suspension, or by passive trapping within the folds and interstices of the test^1,14,15^.

**Figure 1:**
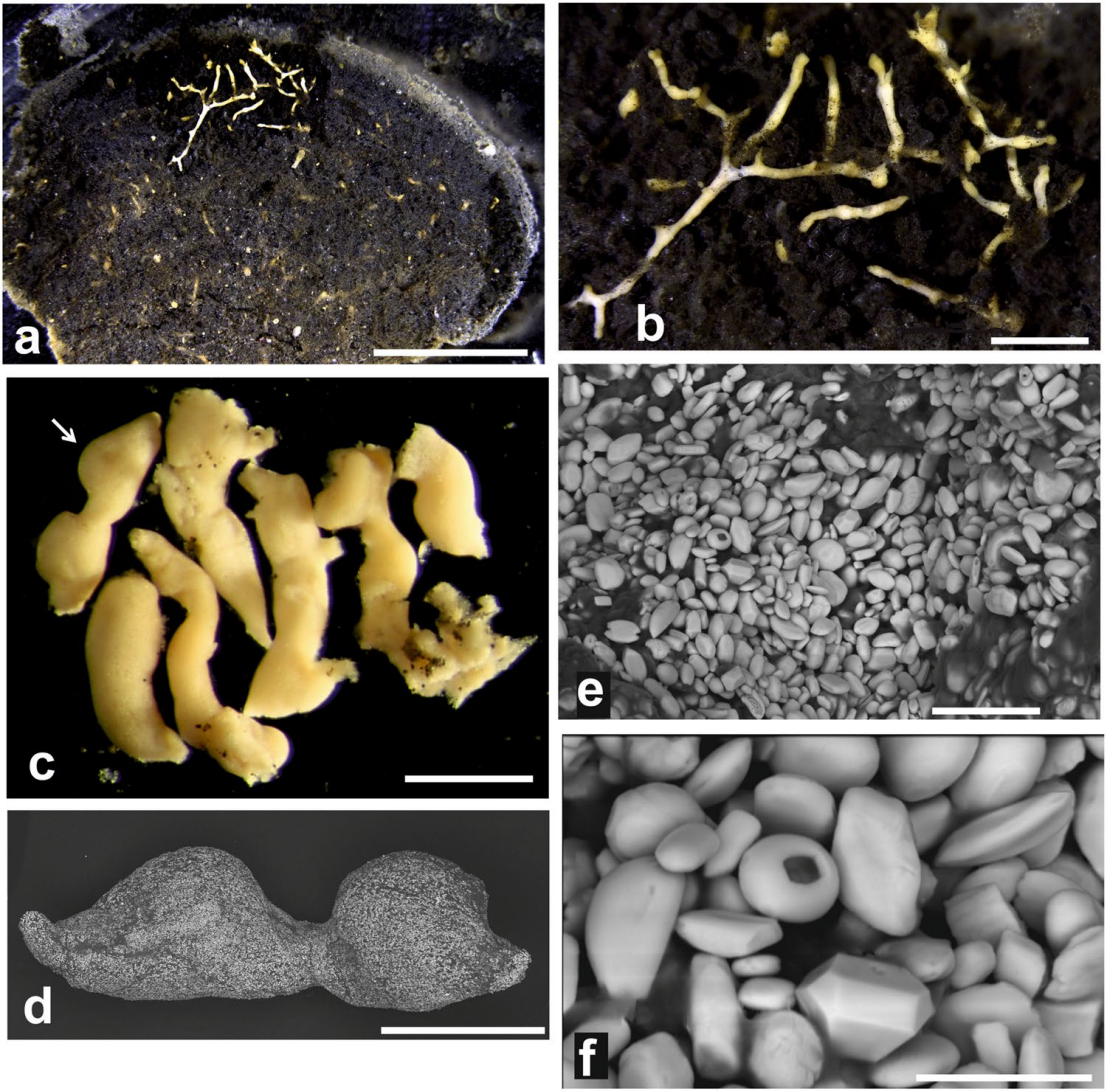
*Psammina* aff. *limbata*, internal features. (**a**–**c**) Light micrographs. (**d**–**f**) Scanning electron micrographs. (**a**) Specimen from Site S02 (BC09); part of the test with a section of the wall removed to show pale granellare strings against a background of dark grey stercomare masses. (**b**) Detail of exposed test interior. (**c**–**f**) Specimen from Site S07 (MC20). (**c**) Fragments of granellare removed from test interior; the arrow indicates the piece shown in figure (**d**). (**d**) Entire fragment full of barite particles. (**e**,**f**) Details of barite particles; note the faceted crystal and the crystal with a rectangular hole in (**f**). Scale bars = 5 mm (**a**), 1 mm (**b**), 0.5 mm (**c**), 200 µm (**d**), 10 µm (**e**), 4 µm (**f**).

The arrangement of the granellare and stercomare in xenophyophores can normally be observed only by breaking open the test. As well as being destructive, this usually reveals only part of the test interior (Fig. 1a,b). Here, we report the first observations of the internal organisation of several xenophyophores obtained by non-destructive micro-CT 3D imaging. This technology uses x-rays to generate 3-dimensional images of the inner structure of objects. It depends on density differences between the components of an object and hence the different degrees to which these components attenuate x-rays. Micro-CT imaging has been used rarely with foraminifera, and then only with calcareous species^16–19^. This is the first such study of a xenophyophore and, as far as we are aware, of any agglutinated foraminiferal species. It is also the first to use this method to visualise the cytoplasm and the test simultaneously; previous studies have examined either the calcareous test but not the cytoplasm^16,18,19^, or the cytoplasm in isolation, following decalcification^17^. The resulting images provide novel information about the relationship between the cytoplasm and test in the case of three large, deep-sea, agglutinated foraminiferal species.

A total of eight xenophyophore specimens belonging to six species were scanned initially, and those that contained well-developed granellare and stercomare were selected for more detailed examination. Our goals are: 1) to determine the extent and distribution of the granellare and stercomare within the agglutinated test and 2) to estimate the relative volumes of these three components of the xenophyophore ‘body’. We also offer some new ideas that may help to explain the conundrum of why xenophyophores concentrate large quantities of heavy minerals within their cells. The specimens included in the present paper are deposited in the Natural History Museum, London, under registration numbers NHMUK PM ZF 7798–7801.

## Results

### *Psammina* aff. *limbata*

**Figure 2:**
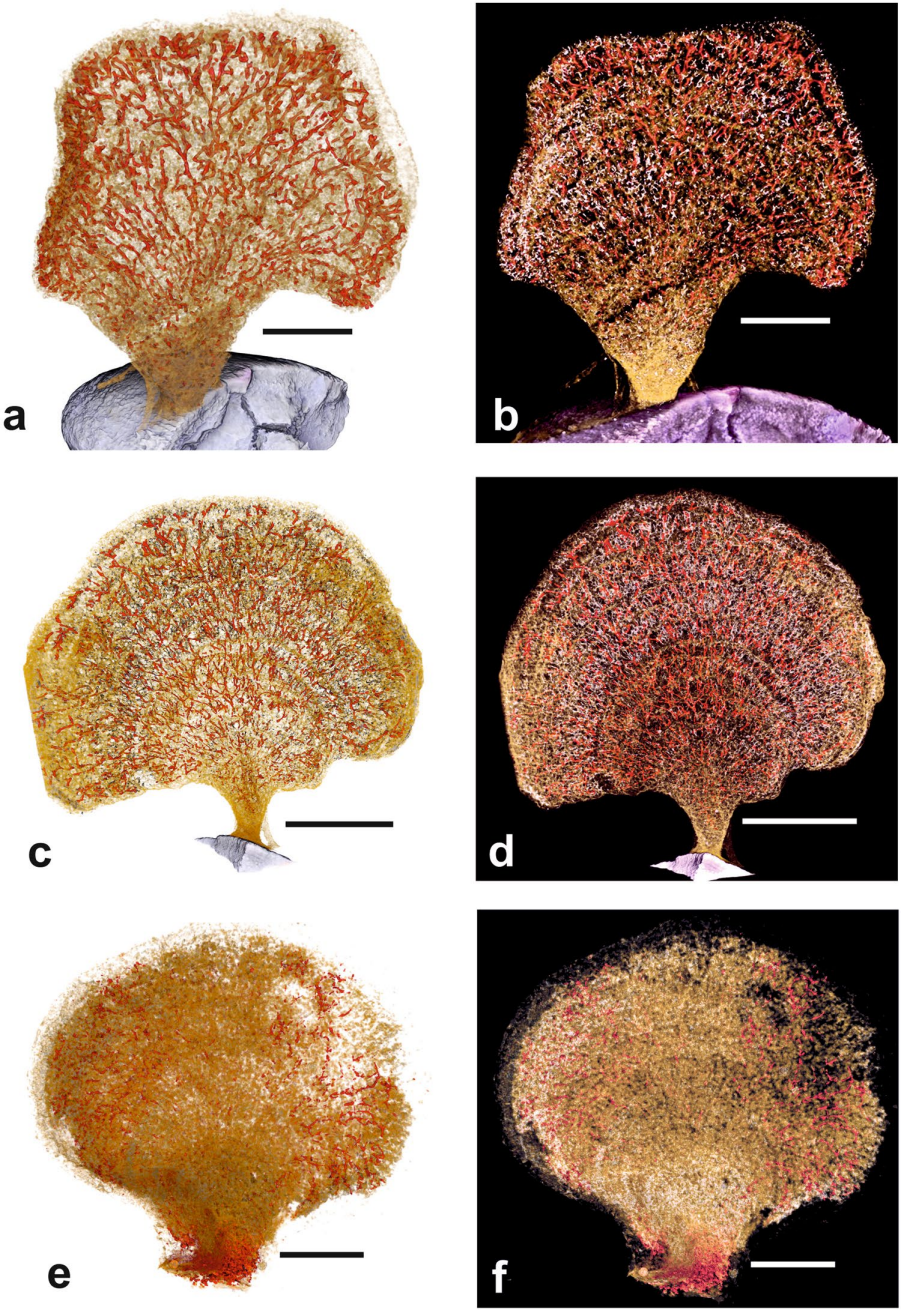
Composite images with light and dark backgrounds in which the three components of the *Psammina* ‘bodies’, granellare (red) stercomare (pale grey) and test material (yellowish-brown), are superimposed. (**a**,**b**) *Psammina* aff. *limbata*, specimen 1. (**c**,**d**) *Psammina* aff. *limbata*, specimen 2. (**e**,**f**) *Psammina* sp. nov. 1. Note that the granellare is more clearly visible against the white background, the stercomare is more clearly visible against the dark background. Scale bars = 10 mm (**a**–**d**), 5 mm (**e**,**f**).

**Figure 3:**
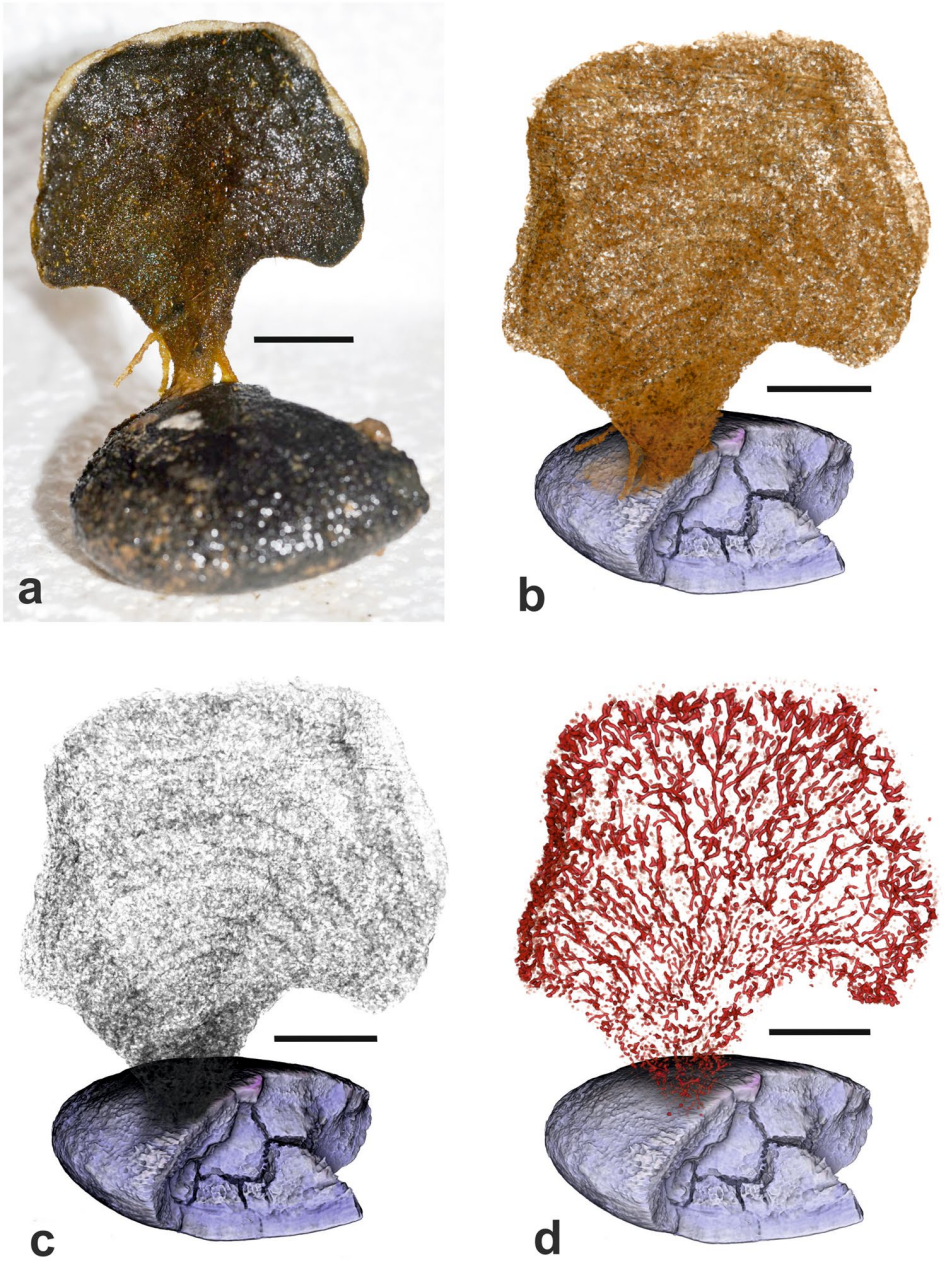
*Psammina* aff. *limbata*, specimen 1 (dry scanned) from Site S10 (BC23). (**a**) Shipboard photograph of test attached to a nodule. (**b**) Medium density material corresponding to the test wall; note the concentric ‘growth lines’. (**c**) Low density material corresponding to the stercomare. (**d**) High-density material corresponding to the granellare. Scale bars = 10 mm.

**Figure 4:**
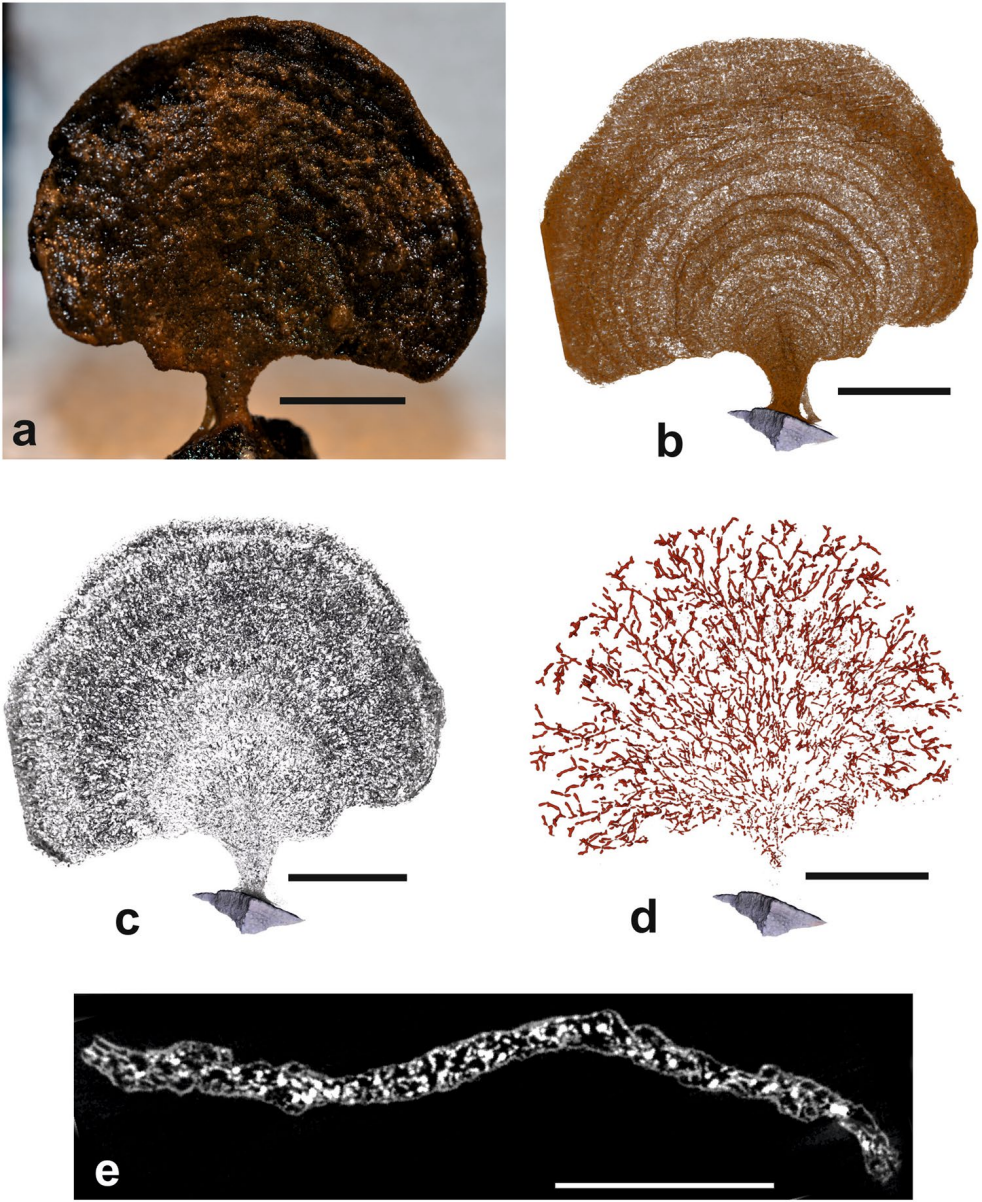
*Psammina* aff. *limbata*, specimen 2 (dry scanned) from Site S11 (BC25). (**a**) Shipboard photograph of test. (**b**) Medium density material corresponding to the test wall; note the concentric ‘growth lines’. (**c**) Low density material corresponding to the stercomare. (**d**) High density material corresponding to the granellare. (**e**) Grey-scale scan section in which the granellare stand out as whitish patches. Scale bars = 10 mm (**a**–**d**), 5 mm (**e**).

Figs 1, 2a–d, 3, 4 and S1; registration numbers NHMUK PM ZF 7798, 7799.

We analysed two intact specimens of this species, both attached to polymetallic nodules and air dried soon after collection without formalin fixation.

#### Light microscope and SEM observations

In both specimens, the test forms a flattened, brownish, fan-shaped structure with a pale rim and a relatively slender basal stalk attached to the nodule surface (Figs 3a and 4a). The test is composed mainly of radiolarian shells and sponge spicules, together with a subordinate proportion of mineral grains. These particles are largely confined to the test wall although some internal spicules are present. Apart from the pale rim, which is more or less empty, the test interior is occupied mainly by dark grey stercomare that forms a closely anastomosing system of branches (180–300 µm or more in diameter) and more irregularly-shaped formations, the boundaries of which are often difficult to discern (Fig. 1a,b). The constituent stercomata appear to be composed of flake-like particles and yield strong aluminium peaks, suggesting a clay mineral composition.

Pale yellowish granellare strands weave between the stercomare branches. In reflected light they stand out prominently against the dark stercomare (Fig. 1a,b). The organic tube that encloses the cytoplasm is not clearly visible and presumably very thin. The cytoplasm is densely packed with crystals (granellae), ranging in size from ~1 to >4 µm (typically 1.5–3.0 µm) in length (Fig. 1e,f). Many of them have rounded, pebble-like shapes but a few are more regular and faceted. The rounded crystals are occasionally interrupted by a deep, clearly-defined depression, sometimes angular in shape. Strong peaks for Ba and S confirm that they are barium sulphate, presumably in the form of barite.

#### Micro-CT scans

Images with the three density components superimposed are shown in Fig. 2a–d. The test material is of mid-density. The scans of both individuals reveal the presence of concentric ‘growth lines’ that are not clearly evident in light photographs of the tests (Figs 3b and 4b). There is no evidence that these features correspond to internal partitions, although mid-density material, most likely sponge spiciules, form an internal mesh in scan slices (Supplementary Fig. S1). The lowest-density component corresponds to the stercomare, which has a granular appearance with a weak radial, fan-like grain superimposed on a vague concentric zonation (Figs 3c and 4c). In specimen 1 the stercomare is well-developed throughout the test, including the stalk, whereas in specimen 2 it is less well developed in the stalk and much denser in the outer part of the semi-circular test compared to the inner part.

The granellare system (the cell body and the organic tube containing it) is the highest density component and is prominently developed. It forms a system of branching strands that pervade the entire test in both specimens. In specimen 1 the strands are relatively thin in the basal stalk and lower central part of the test, where they tend to be aligned, before fanning out in the upper and outer parts of the test (Fig. 3d). The strands are best developed around the test periphery, where they are thicker, denser and branch more frequently, typically ending blindly with somewhat bulbous terminations (Supplementary Fig. S1). They range in width from 80–115 µm in the lower part of the test to 150–190 µm in the centre and 230–300 near the periphery. The granellare system has a similar broadly radial arrangement in specimen 2 (Fig. 4d), but the strands are absent in the lower part of the stalk and spread more evenly across the test in contrast to their strong peripheral development in specimen 1.

### *Psammina* sp. nov. 1

**Figure 5:**
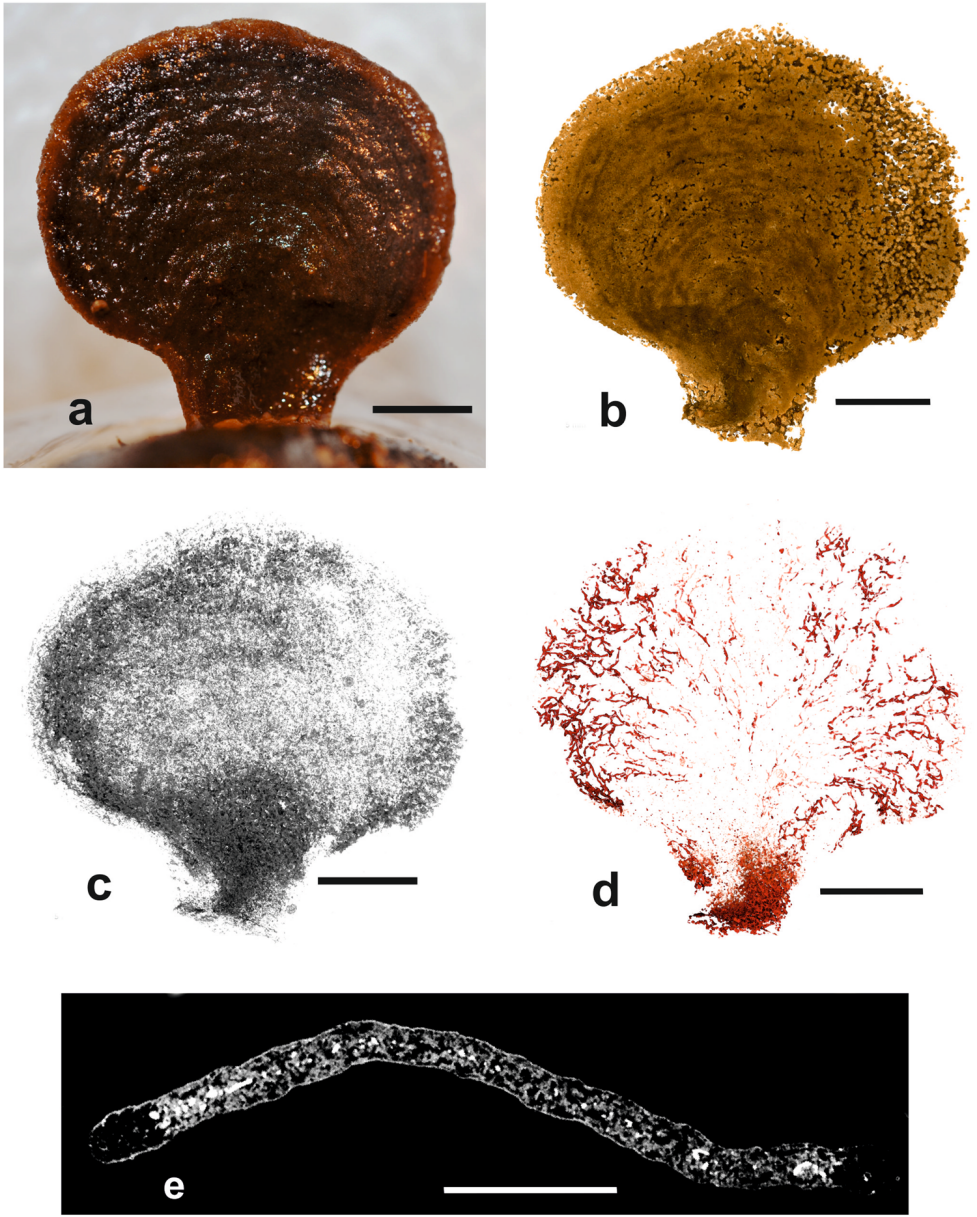
*Psammina* sp. nov. 1 (wet scanned) from Site S07 (BC21). (**a**) Shipboard photograph of test. (**b**) Medium density material corresponding to the test wall; note the vague concentric zonation. (**c**) Low density material corresponding to the stercomare. (**d**) High density material corresponding to the granellare. (**e**) Grey-scale scan section in which the granellare stand out as whitish patches; note the well-defined test wall. Scale bars = 5 mm.

Figs 5 and S2; registration number NHMUK PM ZF 7800.

The test was preserved in 10% formalin and scanned in water. It was found growing on a nodule from which it was detached before preservation.

#### Light microscope and SEM observations

Compared to *P*. aff. *limbata*, the test has a more rounded outline, a shorter and much wider basal stalk (Fig. 5a), and is relatively thicker in cross section. The wall is also finer-grained and includes a much higher proportion of mineral grains than in *P*. aff. *limbata*.

#### Micro-CT scans

Images with the three density components superimposed are shown in Fig. 2e,f. The test wall appears dense with weaker and less regularly developed ‘growth lines’ (Fig. 5b) compared to *P*. aff. *limbata*. The wall is well defined in greyscale scan slices, which also reveal a relatively large amount of material within the test that is of similar density to the wall (Fig. 5e). The low-density stercomare is strongly but unevenly developed. It is particularly dense in parts of the short, wide, basal stalk and to a lesser extent around much of the test periphery with a concentric pattern vaguely discernable (Fig. 5c). The development of the high-density granellare is even more uneven. The branching strands are developed mainly in the two lateral parts of the fan-shaped test (Figs 5d and S2), where they typically measure 100–200 µm in width. In contrast, only sparse, relatively narrow (60–80 µm diameter) discontinuous sections are visible in the central part of the test and near the upper margin (Fig. 5d). Particularly striking are the concentrations of high-density material located in the short, relatively wide stalk. This forms tightly packed interconnected lumps and twisted masses (Supplementary Fig. S2) that are morphologically different from the strands present in the main part of the test. Faint traces of these features extend into the lower part of the fan.

### *Galatheammina* sp

**Figure 6:**
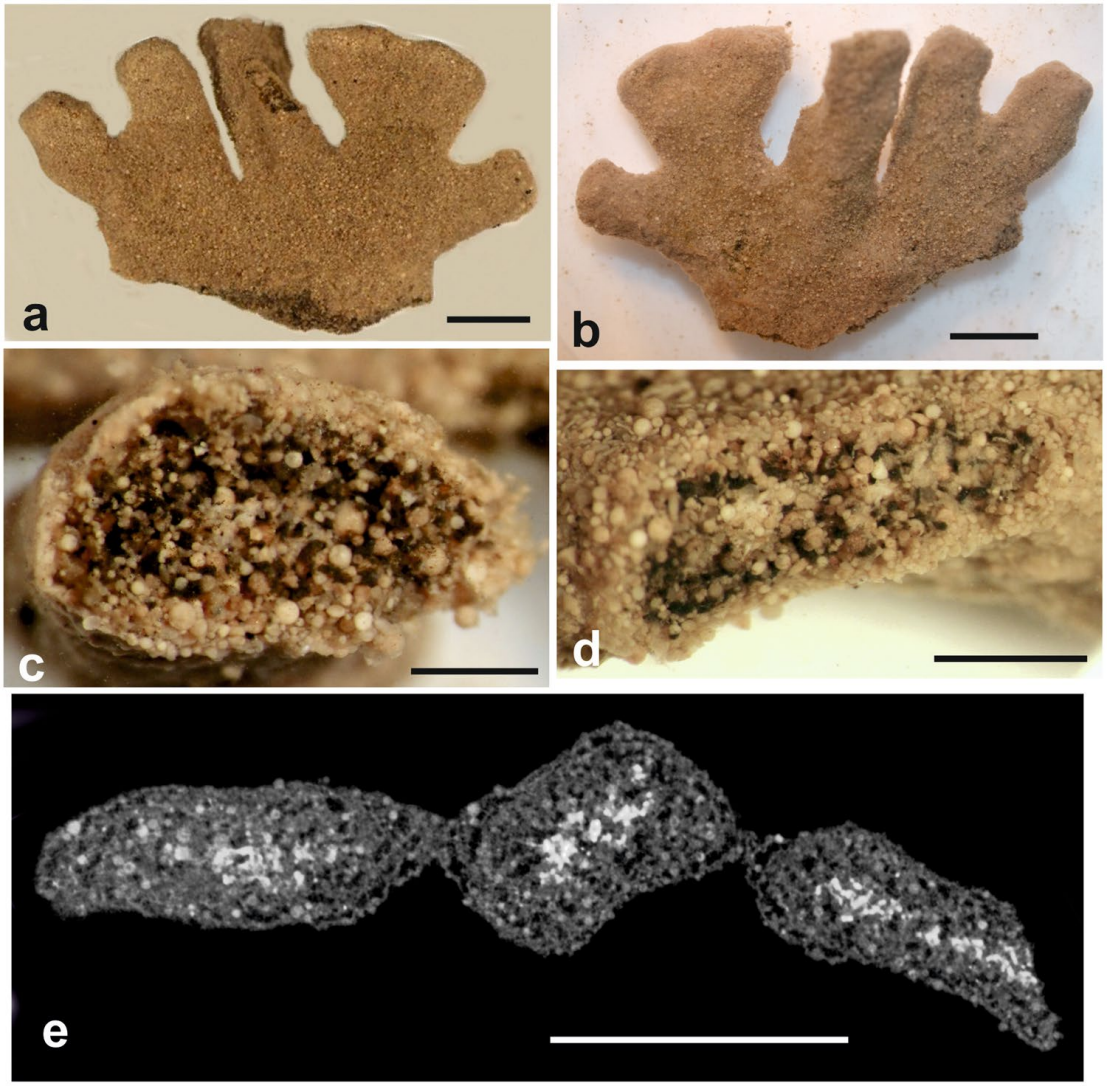
*Galatheammina* sp. (wet scanned) from Site S10 (MC21). (**a**,**b**) Shipboard photographs showing opposite sides of the test as originally found. The outer two lobes were subsequently detached and preserved separately for molecular analysis. (**c**,**d**) Broken sections of the tests showing the internal structure. (**e**) Scan section in greyscale of the test branches with the granellare standing out as whitish patches. Scale bars = 5 mm (**a**,**b**,**e**), 1 mm (**c**,**d**).

**Figure 7:**
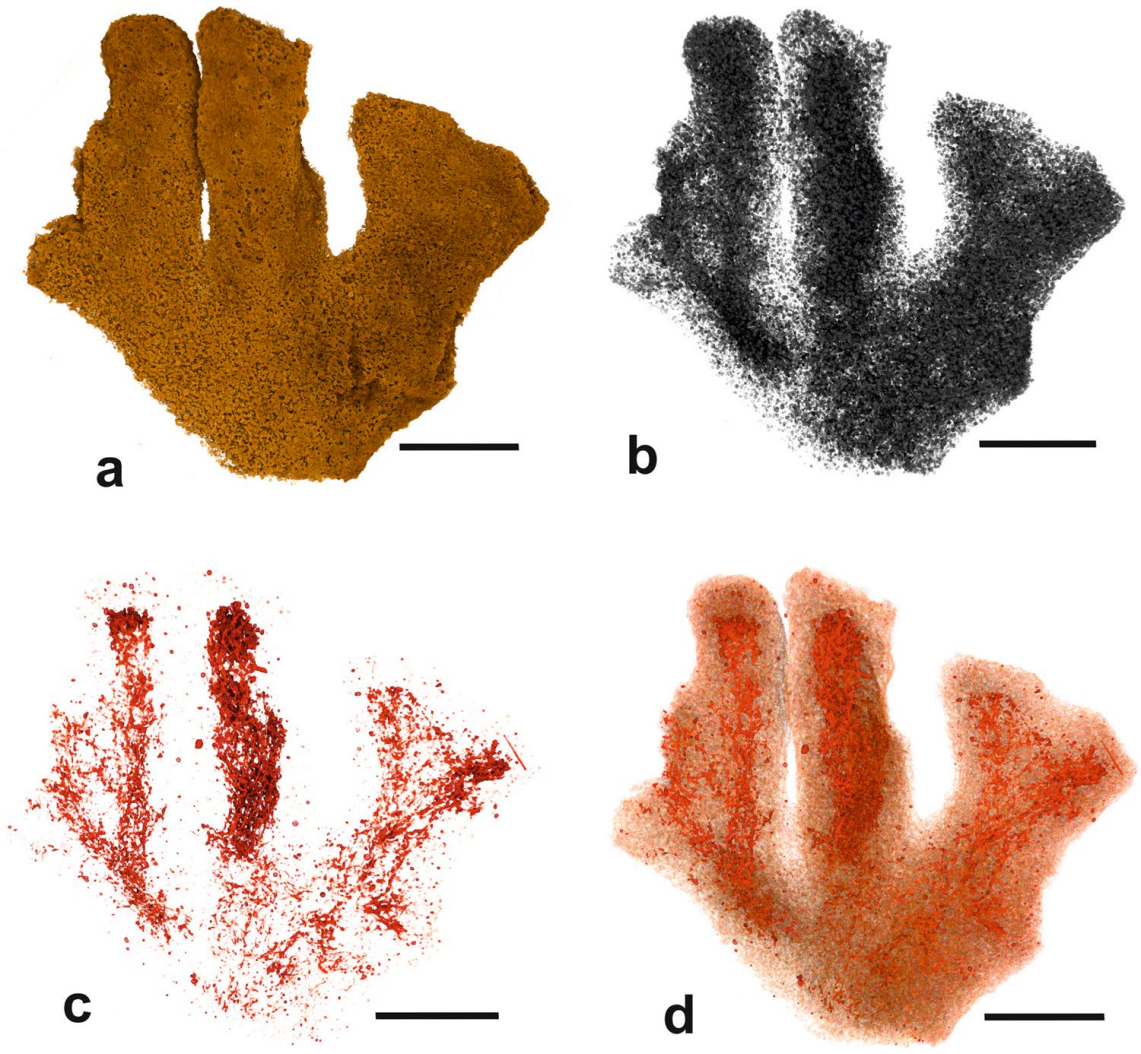
*Galatheammina* sp. (wet scanned) from Site S10 (MC21). (**a**) Medium-density test material corresponding to test particles. (**b**) Low-density material, corresponding to the stercomare and water-filled voids in the test interior. (**c**) High density material corresponding to the granellare. (**d**) Combination of test material (brownish) and granellare (red). Scale bars = 5 mm.

Figs 6 and 7; registration number NHMUK PM ZF 7801.

The single specimen was found lying on the surface of a megacorer sample. It was fixed and preserved in 10% formalin and scanned in water. The proximal part of the test was damaged, possibly during the collection and recovery of the core, and so whether it was originally attached to a polymetallic nodule is unknown.

#### Light microscope and SEM observations

What appears to be the distal end originally had five flattened finger-like processes arising from a plate-like basal part, the lower margin of which is fractured (Fig. 6a,b). The two outer processes were removed soon after the specimen was collected and preserved for molecular analysis (unfortunately unsuccessful because of contamination). Four of the processes were approximately rectangular in shape, the fifth expanded in width towards its outer margin. Tightly packed radiolarians and occasional mineral grains create a fairly uniform outer surface. These agglutinated particles form a thin outer layer that is fairly distinct from the test interior, which includes large numbers of particles, mainly radiolarians similar to those comprising the outer test layer (Fig. 6c,d). The spaces between the internal particles are largely occupied by stercomare, which forms masses of variable width (60–250 µm). Poorly-defined strands of rather diffuse whitish material, presumably part of the granellare system, are sometimes visible on broken surfaces of the test.

#### Micro-CT scanning

Greyscale scan slices (Fig. 6e) are largely consistent with the internal test structure seen in the light microscope. Mid-density particles form an outer layer enclosing material of similar density together with low density material interpreted as stercomare plus water-filled voids. However, in contrast to the light microscope view, where they are difficult to distinguish, the granellare strands stand out clearly in the slices. Seen in lateral view, the test material is dense and featureless (Fig. 7a) and the stercomare pervades the entire structure (Fig. 7b). The high-density granellare is confined to the interiors of the branches (Fig. 7c), forming an intricate web-like mass of strands and filaments concentrated in the axes of the ‘fingers’ of the test (Fig. 7c,d). Individual strands are often narrow (35–70 µm diameter) but widen into broader sections where the system is densely developed, as in the central ‘finger’. The peripheral parts of the test are largely devoid of high-density material, except for some small discrete patches that appear to correspond to agglutinated particles (Fig. 7c).

### Volumetric analysis

In the two scanned tests of *Psammina* aff. *limbata*, the low-density stercomare (and voids between the stercomare masses), occupy most (72.4% and 82.4%) of the volume of the entire xenophyophore ‘body’, with the test material accounting for most of the remainder (14.7% and 22.5%) (Table 1). Despite their visual prominence, the strands of the granellare system contribute only 2.9% and 5.1% of the total volume. The actual volumes of these three components vary substantially between the two specimens, particularly in the case of the granellare, where the volume occupied in specimen 1 is twice that occupied in specimen 2. The relative proportions of test material, stercomare, and granellare are similar in *Psammina* sp. nov. 1, although their actual volumes are much less than in either of the two *P*. aff. *limbata* specimens. More than half (58.8%) of the total ‘body’ volume of *Galatheammina* sp. corresponds to the test material and the actual volume of the test (331 mm^3^) is also higher than in any of the *Psammina* specimens (Table 1). On the other hand, the stercomare (39.5%) and granellare (1.7%) make a correspondingly lower contribution in both absolute and relative terms.

**Table 1:** Volumes of different components of xenophyophore ‘bodies’, determined from micro-CT scan data.

Species	Condition	Test material (mm^3^)	Granellare (mm^3^)	Stercomare and empty space (mm^3^)
*P*. aff. *limbata* specimen 1	Dry	321 (22.5%)	72.6 (5.1%)	1031 (72.4%)
*P*. aff. *limbata* specimen 2	Dry	175 (14.7%)	33.9 (2.9%)	980 (82.4%)
*Psammina* sp. nov. 1	Wet	98.5 (18.0%)	15.3 (2.8%)	433 (79.2%)
*Galatheammina* sp.	Wet	331 (58.8%)	9.45 (1.7%)	222 (39.5%)

Note that the granellare comprises the cell body, the intracellular barite crystals, and the organic tube that surrounds the cytoplasm. The tube is very thin, almost invisible in the analysed species and of negligible volume. However, the barite crystals (granellae) are numerous and occupy a substantial amount of space. As a rough estimate, we suggest that ~50% of the granellare volume is occupied by these crystals.

## Discussion

This study demonstrates that valuable information on the three-dimensional internal structure of xenophyophores can be obtained from micro-CT imaging. In particular, the granellare (mainly the cytoplasm and intracellular barite crystals; the enclosing tube has an insignificant volume, at least in *P*. aff. *limbata*) stands out clearly from other test components. This is surprising given that the cytoplasm itself has a relatively low density, around 1.02 g.ml^−1^ close to that of water^20^, and therefore should not be imaged effectively in CT scans. Nevertheless, the granellare system is clearly visible as high-density material in our images of dry and wet xenophyophores. This must be attributable to the large concentrations within the granellare of the heavy mineral barite (Fig. 1d–f), which has a density of 4.48.

The two dried specimens of *Psammina* aff. *limbata* (Figs 3a and 4a) differ in some respects from *P*. *limbata* from the Russian license area in the central CCZ^21^. In particular, they are much less strongly curved around a vertical axis and lack the extensive system of root-like structures that are a striking feature of the holotype. The other *Psammina* species, which was scanned in water (Fig. 5), has a thicker, more rounded test than the two dried specimens as well as a much wider stalk. It almost certainly represents a distinct species.

### Organisation of the cell body

Schultze^2^ illustrated examples of branching granellare strands that had been isolated from specimens of the xenophyophore *Psammetta erythrocytomorpha*. Given the difficulty of extracting these delicate structures intact, Schultze’s illustrations probably only represent part of the granellare. Nevertheless, they give a good impression of the appearance of the system in a typical xenophyophore and are broadly similar to granellare morphologies revealed by our CT scans of *Psammina* aff. *limbata* and *Psammina* sp. nov. 1. In these species the branches of the cell body fan out across the test interior and seem well suited to delivering pseudopodia to all parts of the test rim, thereby maximizing the chances of intercepting suspended food particles^14^. The granellare in *Galatheammina* sp. has a somewhat different appearance, comprising a tightly-meshed cobweb of finer strings that are concentrated in the more central part of the test.

The granellare in the two dried specimens of *Psammina limbata* give the impression of being a continuous system, but with fairly numerous gaps (see particularly Supplementary Fig. S1). These gaps most likely originated from shrinkage of the cytoplasm during drying, but could also possibly be original features. A shipboard examination of the test interior of freshly collected, unfixed specimens of another xenophyophore species, *Aschemonella monile*, recently described from the eastern CCZ, revealed what appeared to be isolated sections of granellare, in some cases linked by diffuse wisps of cytoplasm^22^. The granellare of *Psammina* sp. nov. 1, which was scanned wet (i.e., without ever having been dried), displays similar discontinuities as well as some other interesting features. The granellare is prominently developed in the lateral parts of the test but very faint in the centre and along the middle part of the upper margin. This could reflect much lower concentrations of barite crystals in these areas, or possibly the inability of the scan to resolve the granellare strands where they are narrower. Most curious is the strong development of high density material (presumably barite-bearing cytoplasm), as well as low-density stercomare, in parts of the stalk. Why cytoplasm should be concentrated at the base of the test is unclear.

### Estimation of granellare volume and biomass

The granellare branches are generally wider in *Psammina* aff. *limbata* and *Psammina* sp. nov. 1 (80–300 µm) than in *Galatheammina* sp. (typically <100 µm); the former lie at the upper end of the range of width values for xenophyophore granellare summarised by Tendal^1^ while the latter lie towards the lower end. *Aschemonella monile* also has relatively wide (105–190 µm) granellare branches^22^, as well as a hollow interior devoid of internal test particles. In this latter respect it is more similar to *P*. aff. *limbata*, in which internal particles are relatively sparse, than to *Galatheammina* sp. This might suggest that species in which internal particles are absent or few in number have a more voluminous granellare system than species in which the interior is packed with particles. However, the data compiled by Tendal^1^ suggests that this is not the case; two species of *Psammina* (*P*. *nummulina* and *P*. *globigerina*) have narrow (30–60 and 40–90 µm, respectively) granellare branches while the species with the widest branches (~330 µm, *Psammetta ovale*) has a solid test.

Tendal^1^ concluded that the ‘granellare constitute only a small part of the total body volume; the largest part of a xenophyophore is dead matter, consisting of stercomare and xenophyae’. Levin and Gooday^20^ attempted to quantify this statement. They arrived at rough subjective estimates of the granellare volume as a percentage of the test volume in 12 Pacific xenophyophores belonging to 11 species, based on the visual inspection of the specimens by 5 different people. Most of the modal values were ≤1%, the lowest being only 0.01% (in *Galatheammina* sp. and *Stannophyllum zonarium*), but reached 2–5% in the case of a *Psammina* species. The present study demonstrates that micro-CT 3D imaging is a good method for accurately measuring the relative volumes of the granellare and other components of the xenophyophore ‘body’. In terms of percentages, our results are broadly consistent with Levin and Gooday’s estimates for their *Psammina* species^20^, although higher, in some cases much higher (Table 1), than those of the other species they considered. The CT scans also provide precise data on the actual volume of the granellare, from which the biomass of individual specimens may be derived using a conversion factor of 1 ml cytoplasm = 1.02 g wet wt^20^. The availability of such data for a range of species, combined with photographic surveys of the seafloor in areas such as the CCZ where xenophyophores are abundant^8,9^, could form a basis for estimating the contribution of xenophyophores to megafaunal biomass. However, it is important to note that the granellare shows up well in CT scans because the cytoplasm contains large numbers of dense barite crystals (Fig. 1d–f). The proportion occupied by crystals cannot be derived from CT scan data, but as a rough estimate we suggest that it could amount to around half of the granellare volume. This needs to be taken into account when estimating cellular biomass.

### Possible function of the granellae

The presence of numerous granellae in the cytoplasm of xenophyophores has provoked considerable interest and speculation regarding their origin and function. For many years the question of whether these crystals are taken up from the sediment or secreted within the cytoplasm remained unresolved^1,11^. However, as mentioned above, the dense concentrations of titanium-bearing minerals in the granellare of a xenophyophophore from the Nazaré Canyon (Portuguese margin)^12^ provides a strong indication that xenophyophores accumulate heavy minerals. Detrital titanium-bearing minerals derived from land are readily available in submarine canyons, whereas barite is the only heavy mineral that is common in open-ocean settings^23^. A sedimentary origin is also consistent with the close similarity in terms of size (from ~1 to several microns) and morphology (generally more or less rounded, sometimes subangular or angular, occasionally with a well-defined depression) between the granellae of xenophyophores and barite crystals of pelagic origin found in modern and ancient deep-sea sediments (compare Fig. 1e,f herein with Fig. 1a–d in Paytan *et al*.^24^). The ability of agglutinated foraminifera to select particles of particular size, shape or density^25^, including barite grains^26^, for incorporation into their tests is well known. For these reasons we assume that the barite is acquired from the environment rather than being precipitated within the cell. What purpose is served by packing the cytoplasm with massive quantities of these heavy minerals, however, remains an open question.

Some small monothalamids, notably flask-shaped saccamminids in the genus *Psammophaga*, also collect heavy minerals (magnetite, titaniferous magnetite, ilmenite, rutile, zircon, among others) within their cytoplasm^27–29^. Since the crystals are generally concentrated inside the aperture, it is possible that they serve to ballast this end of the test so that the aperture is directed towards the sediment^30^. However, such a function seems implausible in the case of large, relatively heavy xenophyophore tests, which have no obvious need for additional ballast. The suggestion that saccamminids such as *Psammophaga* are feeding on bacteria adhering to heavy-mineral grains^28^ could apply to xenophyophores. The barite crystals found in deep-sea sediments are believed to form inside organic-rich aggregates in the upper water column (0–200 m), subsequently sinking to the seafloor following the decomposition of the organic matrix^31,32^. It is possible that they acquire some nutritional value (e.g., from adhering bacteria) during their 4-km passage through the water column. However, a more likely explanation for why xenophyophores concentrate barite may be that the granellae are used to shape their cells, making them bulkier and more rigid. This would help to support and maintain the extensive system of branching cellular strands that ramify the entire test, as revealed by our micro-CT images (Figs 3–5d and 7c,d). This seems particularly plausible in the case of *Psammina* aff. *limbata*. When granellare fragments from this species were dried onto SEM stubs, they underwent relatively little shrinkage because their shape was supported by the densely packed granellae (Fig. 1d). The bulking out of the granellare with barite crystals recalls the retention by xenophyophores of large stercomare masses, creating a ‘body’ that has a large volume but contains a relatively low cytoplasmic biomass^1,33^. This kind of ‘high volume low biomass’ organisation might be an advantage for organisms living in food-poor abyssal deep-sea environments.

The accumulation of granellae could impart other benefits. If these very large cells consisted entirely of active organelles, their maintenance would place a heavy metabolic burden on the xenophyophores. Packing the cell bodies with dense crystals, leaving the extremities and pseudopodia as the main active regions, may serve to lighten the metabolic load. In this respect, there are possible parallels between xenophyophores and gigantic bacteria, e.g. the *Thiomargarita* sulphur-oxidising bacterium. The spherical cells of these prokaryotes are typically 100–300 µm in size but can reach diameters of up to 750 µm^34^. However, some 98% of their volume is occupied by a liquid-filled vacuole; the metabolically active cytoplasm is confined to a thin outer layer and contains numerous inert sulphur granules, further reducing its volume. In *Psammina* aff. *limbata*, and other xenophoyphores such as *Aschemonella ramuliformis*^35^, *A*. *monile*^22^ and *Nazareammina tenera*^11^, it is the granellae that occupy a large proportion of the cell volume. It should be noted, however, that barite crystals appear to be either absent or rare in a few species^1,36^ and, as mentioned above, there are indications that crystal densities could be reduced in the granellare strands towards the centre of the wet-scanned *Psammina* sp. nov. 1 (Fig. 5d). Another point to consider is that stercomata (waste pellets) are sometimes composed entirely of granellae, suggesting that they are expelled from the cell as well as accumulating within it^1^. Finally, there is no clear answer to the question of why xenophyophores should select heavy minerals, notably barite, as granellae, rather than lighter particles such as feldspar and volcanic glass fragments, both of which are incorporated into the tests of several *Psammina* species at our sampling sites (unpublished observations). In this regard it is interesting to note that high concentrations of mercury have been reported from the granellare of *Shinkaiya lindsayi*^36^, suggesting that some xenophyophores have an affinity for heavy metals as well as for heavy minerals.

## Materials and Methods

### Sample collection and preservation

We used CT imaging to examine 2 specimens of *Psammina* aff. *limbata* Kamenskaya, Gooday, Tendal 2015, one specimen of *Psammina* sp. nov. 1, and one specimen of an undescribed species assigned to *Galatheammina*. They were collected during the second ABYSSLINE cruise (AB02: R/V *Thomas G Thompson* cruise TN319; 12^th^ February to 25^th^ March, 2015) in the OMS Stratum, a 30 × 30 km area of seafloor centred around 12°8.2′N, 117°17.7′W (Smith *et al*., 2015). Station data are summarised in Supplementary Table S1. Soon after collection, specimens were photographed extensively using either a hand-held Nikon D3100 SLR digital camera fitted with a Nikon 62 mm macro lens or, for details, a Canon 60D SRL digital camera attached to an Olympus SZX7 microscope. The tests of *P*. aff. *limbata* were washed in fresh water and air dried. The two other specimens (*P*. sp. nov. 1 and *Galatheammina* sp.) were fixed and preserved in 10% formalin buffered with borax. Two additional formalin-fixed specimens of *P*. aff. *limbata* from the OMS Stratum were used to study internal test structure using light and scanning electron microscopy.

### Microscopy

Light images were obtained either at sea, as described above, or in the laboratory using a Leica M205 C stereomicroscope equipped with a Leica DFC 450 C camera. Cytoplasm extracted from *Psammina* aff. *limbata* was examined using scanning electron microscopy (SEM). Fragments of the granellare were air-dried onto an SEM stub and examined with a FEI Quanta 650 FEG SEM in low vacuum under the following conditions: Accelerating voltage of 10 kV, BSE detector, chamber pressure 40 Pa.

### Micro-CT imaging

During micro-CT scans over 2,000 X-ray projections are collected through a 360° rotation of the sample, the grayscale values in these images representing the X-ray attenuation within the sample (a proxy for density). For the present study, the selected xenophyophores were scanned using the Nikon HMX ST 225 micro-CT scanner (Nikon Metrology, Tring, UK) at the Natural History Museum, London. This system is equipped with a detector panel (2000 × 2000 pixels) with a maximum resolution (voxel size) of 5 μm and a maximum energy of 225 kV. Specimens were scanned either dry or immersed in water in straight-sided polypropylene jars, using a tungsten reflection target. Individual scanning parameters are summarised in Supplementary Table S2. Images acquired during the scanning process were subsequently reconstructed using the software CT Pro (Nikon Metrology, Tring, UK), which employs a modified version of the Feldkamp *et al*. back-projection algorithm^37^. This generated a stack of grayscale TIFF slice images, which were then imported into the Drishti software suite^38^. In Drishti a 3D rendering of the specimens was produced, with different transfer functions created for xenophyophore test material, granellare and stercomare based upon the relative X-ray attenuation (a proxy for density) of each component. Thresholds were determined based on a histogram of the grayscale values for the entire reconstructed data. The peaks in the data are used for binning the data into three distinct materials: granellare, stercomare (including some air/water-filled space), and test material. The distributions of these density-based components correspond well with the granellare, stercomare, and test material, as seen by light microscopy. The slice images were then imported into Avizo (version 9.0; Visualisation Centre Group) for segmentation of individual anatomical elements based on grayscale values (relative X-ray absorption) to calculate the volume of each element.

### Data availability

The four specimens on which this study is based are deposited in the Natural History Museum, London, under registration numbers NHMUK PM ZF 7798–7801. The raw micro-CT data can be accessed via the public Data Portal of the Natural History Museum, London (http://data.nhm.ac.uk).

## Electronic supplementary material

Supplementary material
