# Supplementary material for: Micro-CT 3D imaging reveals the internal structure of three abyssal xenophyophore species (Protista, Foraminifera) from the eastern equatorial Pacific Ocean
Source: Sci Rep. 2018 Aug 14;8:12103. doi: 10.1038/s41598-018-30186-2 (PMC6092355; doi:10.1038/s41598-018-30186-2)
Supplement: Supplementary file 1 — Supplementary material [file 41598_2018_30186_MOESM1_ESM.pdf]

Micro-CT 3D imaging reveals the internal structure of three abyssal xenophyophore species (Protista, Foraminifera) from the eastern equatorial Pacific Ocean

Andrew J Gooday, Dan Sykes, Tomasz Góral, Mikhail V Zubkov, Adrian G. Glover

Supplementary Information

Supplementary Figure S1.
Psammina aff. *limbata,* specimen 1 (dry) from Site S10 (BC23).

Supplementary Figure S2.
Psammina sp. nov. 1 (wet) from Site S07 (BC21).

Supplementary Table S1.
Station data for samples collected during the AB02 cruise that yielded the studied xenophyophores

Supplementary Table S2.
Micro-CT imaging parameters for the scanned xenophyophore specimens.

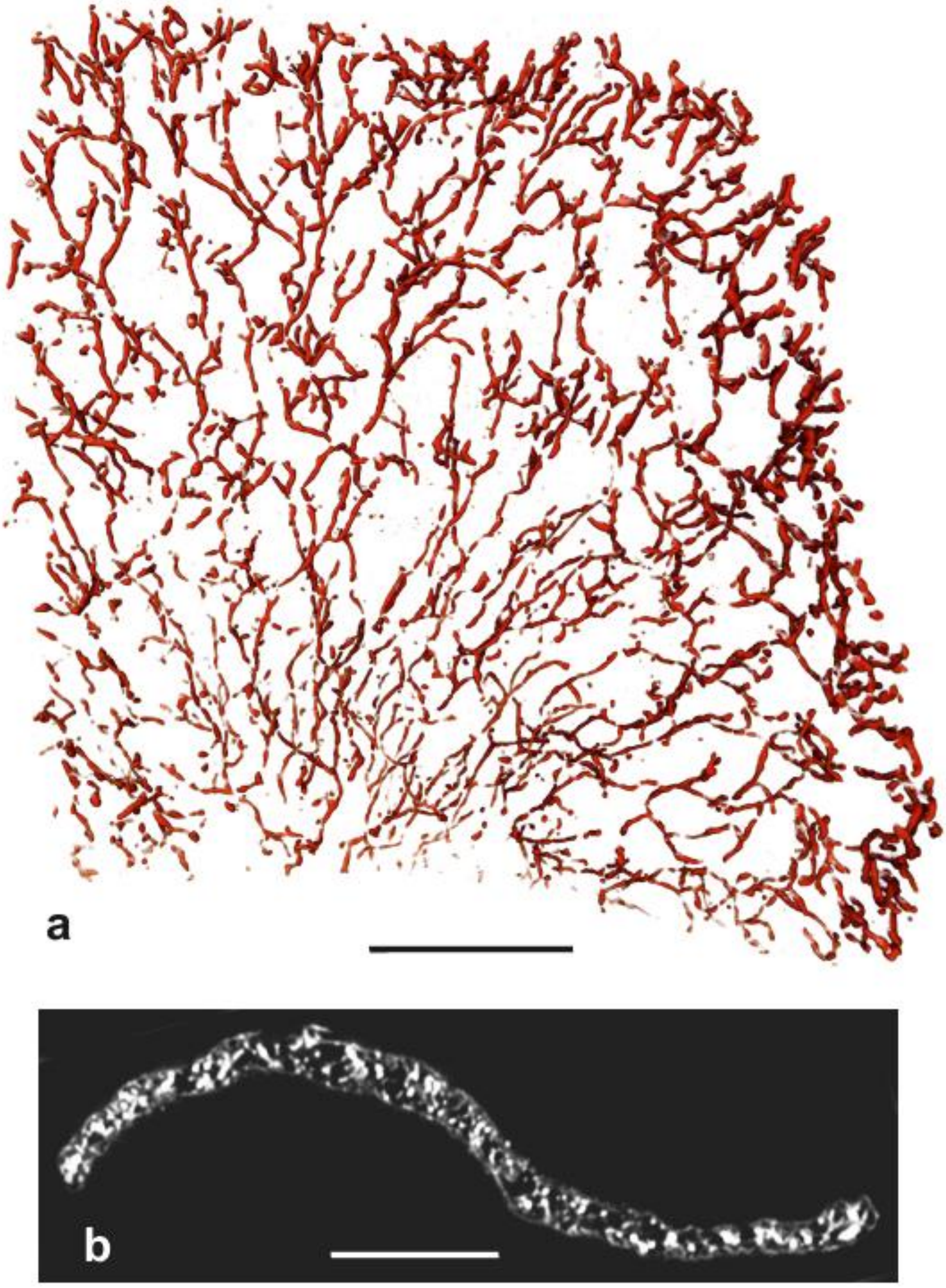

Supplementary Figure S1. *Psammina* aff. *limbata*, specimen 1 (dry) from Site S10 (BC23). (**a**) High density material corresponding to the granellare (the cell body). (**b**) Slice through the test; the outer wall is clearly defined, the bright areas are cross sections of the granellare and the fainter structures are probably internal agglutinated sponge spicules. Scales = 5 mm.

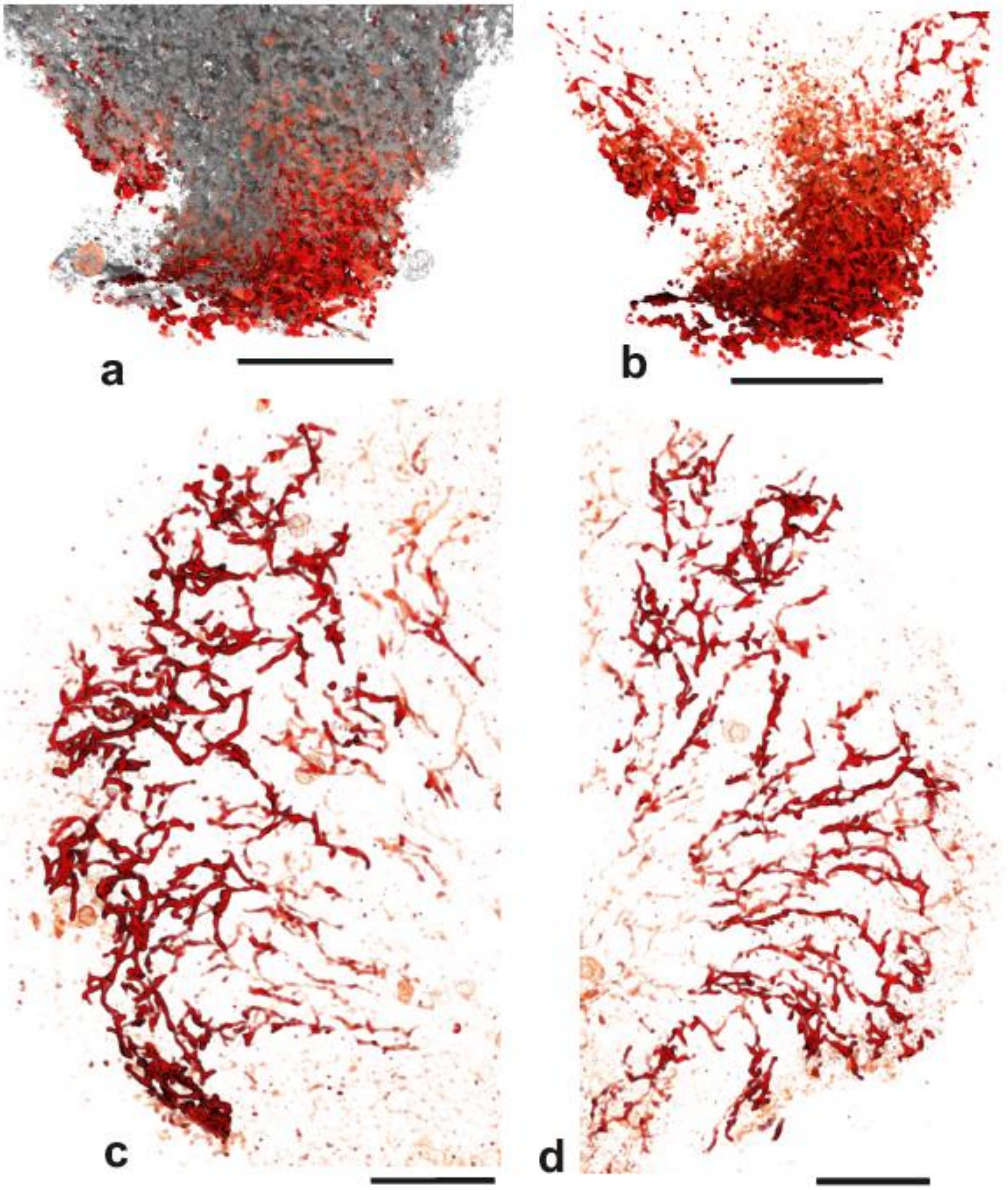

Supplementary Figure S2. *Psammina* sp. nov. 1 (wet) from Site S07 (BC21). (**a**) Base of test including the short stalk showing mass of high density material (red) corresponding to the granellare and medium density material (grey) corresponding to the stercomare. (**b**) Same area showing only the high density material. (**c, d**) The two sides of the test showing the well-developed granellare. Scale bars = 2.5 mm.

Supplementary Table S1. Station data for samples collected during the AB02 cruise that yielded the studied xenophyophores. The specimens from the BC09 and MC20 deployments were used for light and scanning electron microscopy. BC = Sample collected using an USNEL box corer; MC = Sample collected using a Megacorer equipped with 10-cm-diameter core tubes.

**Site**	**Deployment**	**Latitude N**	**Longitude W**	**Depth (m)**	**Species**
S02	BC09	12°04.912′	117°10.691′	4070	*P.* aff. *limbata*
S07	BC21	12°08.156'	117°12.900'	4054	*Psammina* sp. nov. 1
S07	MC20	12°08.163'	117°12.898'	4054	*P.* aff. *limbata*
S10	MC21	12°03.279'	117°15.095'	4096	*Galatheammina* sp.
S10	BC23	12°03.277'	117°15.104'	4100	*P.* aff. *limbata* spec. 1
S11	BC25	12°00.559'	117°22.818'	4141	*P.* aff. *limbata* spec. 2

a

Supplementary Table S2. Micro-CT imaging parameters for the scanned xenophyophore specimens.

**Specimen**	**kV**	**μA**	**Exposure (ms)**	**Number of projections**	**Filter**	**Voxel size (μm)**
Psammina aff. *limbata* 1	180	200	708	3142	None	39
Psammina aff. *limbata* 2	110	270	708	3142	0.1 mm copper	21
Psammina sp. nov. 1	180	200	708	3142	None	16
Galatheammina sp.	110	270	708	3142	None	13
